# *Apolipoprotein E* E3/E4 genotype is associated with an increased risk of premature coronary artery disease

**DOI:** 10.1186/s12872-024-04021-8

**Published:** 2024-07-10

**Authors:** Youqian Li, Wei Zhong, Changjing Huang, Junyin Peng, Hanlin Li

**Affiliations:** 1 Center for Cardiovascular Diseases, Meizhou People’s Hospital, Meizhou Academy of Medical Sciences, No. 63 Huangtang Road, Meijiang District, Meizhou, China; 2 Guangdong Provincial Engineering and Technology Research Center for Molecular Diagnostics of Cardiovascular Diseases, Meizhou People’s Hospital, Meizhou Academy of Medical Sciences, Meizhou, China

**Keywords:** Premature coronary artery disease, Apolipoprotein E, Coronary artery disease, Polymorphism

## Abstract

**Objective:**

Dyslipidemia is one of the causes of coronary heart disease (CAD), and apolipoprotein E (APOE) gene polymorphism affects lipid levels. However, the relationship between *APOE* gene polymorphisms and premature CAD (PCAD, male CAD patients with ≤ 55 years old and female with ≤ 65 years old) risk had different results in different studies. The aim of this study was to assess this relationship and to further evaluate the relationship between *APOE* gene polymorphisms and PCAD risk in the Hakka population.

**Methods:**

This study retrospectively analyzed 301 PCAD patients and 402 age matched controls without CAD. The *APOE* rs429358 and rs7412 polymorphisms were genotyped by polymerase chain reaction (PCR) -chip technique. The distribution of *APOE* genotypes and alleles between the case group and the control group was compared. The relationship between *APOE* genotypes and PCAD risk was obtained by logistic regression analysis.

**Results:**

The frequency of the *APOE* ɛ3/ɛ4 genotype (18.9% vs. 10.2%, *p* = 0.001) and ε4 allele (11.1% vs. 7.0%, *p* = 0.007) was higher in the PCAD patients than that in controls, respectively. PCAD patients with ɛ2 allele had higher TG level than those with ɛ3 allele, and controls carried ɛ2 allele had higher HDL-C level and lower LDL-C level than those carried ɛ3 allele. Regression logistic analysis showed that BMI ≥ 24.0 kg/m^2^ (BMI ≥ 24.0 kg/m^2^ vs. BMI 18.5–23.9 kg/m^2^, OR: 1.763, 95% CI: 1.235–2.516, *p* = 0.002), history of smoking (Yes vs. No, OR: 5.098, 95% CI: 2.910–8.930, *p* < 0.001), ɛ3/ɛ4 genotype (ɛ3/ɛ4 vs. ɛ3/ɛ3, OR: 2.203, 95% CI: 1.363–3.559, *p* = 0.001), ε4 allele (ε4 vs. ε3, OR: 2.125, 95% CI: 1.333–3.389, *p* = 0.002), and TC level (OR: 1.397, 95% CI: 1.023–1.910, *p* = 0.036) were associated with PCAD.

**Conclusions:**

In summary, BMI ≥ 24.0 kg/m^2^, history of smoking, *APOE* ɛ3/ɛ4 genotype, and TC level were independent risk factors for PCAD. It means that young individuals who are overweight, have a history of smoking, and carried *APOE* ɛ3/ɛ4 genotype had increased risk of PCAD.

## Introduction

Cardiovascular disease (CVD) is one of the major disease burdens, and cardiovascular disease has become the leading cause of death in China [1, 2]. Coronary artery disease (CAD) is a heart disease caused by the stenosis, spasm and obstruction of the artery lumen caused by atherosclerosis, which leads to myocardial ischemia, hypoxia or necrosis [3, 4]. CAD is a serious cardiovascular disease with high morbidity and mortality, and is one of the serious disease burdens [5, 6]. Although CAD is mainly in the elderly, the trend of CAD is younger at present [7, 8]. According to Third Report of the National Cholesterol Education Program (NCEP) Expert Panel on Detection, Evaluation, and Treatment of High Blood Cholesterol in Adults (Adult Treatment Panel III), premature coronary artery disease (PCAD) refers to the occurrence of CAD when men ≤ 55 years old and women ≤ 65 years old [9]. Compared with non-PCAD patients, PCAD patients often have no precursor symptoms, the course of the disease is rapid, and acute coronary syndrome is often the clinical manifestation [10]. Therefore, it is particularly important to understand the risk factors of PCAD and avoid the risk factors.

Lipid metabolism disorders are closely related to CAD, and apolipoprotein plays an important role in lipid metabolism. Apolipoprotein E (ApoE) is one of the important apolipoproteins involved in lipid metabolism and cholesterol metabolism, cholesterol transport and redistribution, immune regulation, cell proliferation and differentiation [11–14]. ApoE is an important component of very low density lipoprotein cholesterol (VLDL-C) and high density lipoprotein cholesterol (HDL-C), and is a ligand protein of chylomicron and VLDL receptor in hepatocytes, which can bind to chylomicron and VLDL receptor with high affinity. ApoE is also a cofactor of lipoprotein metabolic enzymes, which can mediate the intracellular phagocytosis and degradation of lipoproteins [15, 16]. The ApoE encoding gene *APOE* is located on chromosome 19q13.2 and contains four exons and three introns [16]. There are several single-nucleotide polymorphisms (SNPs) in *APOE* gene, the most important of which are rs429358 and rs7412, which form three allelic subtypes of *APOE*, namely ε2, ε3 and ε4 [17, 18], and six common *APOE* genotypes: ɛ2/ɛ2, ɛ2/ɛ3, ɛ2/ɛ4, ɛ3/ɛ3, ɛ3/ɛ4, and ɛ4/ɛ4 [19, 20]. The mechanism of lipid difference caused by different *APOE* genotypes may be related to the differences in the ability of ApoE to bind to receptors and the rate of lipoprotein metabolism [21].

Studies have showed that *APOE* gene polymorphism was established to be related to PCAD. The *APOE* ɛ4 allele may be associated with an increased risk of PCAD due to elevated levels of total cholesterol (TC) and low density lipoprotein (LDL-C) [22]. The results of a meta-analysis suggested that the *APOE* ɛ2 allele may be a risk factor for PCAD in Asians and a protective factor in Caucasians [23]. The study by Petrovic D et al. found that *APOE* gene polymorphism was not an independent risk factor for PCAD in the Slovenian population [24]. It can be seen that the results on the relationship between *APOE* gene polymorphism and PCAD are inconsistent, which may be caused by the differences between the different regions, populations, lifestyles and the interaction between some environmental factors and genetic polymorphisms, and sample size of the subjects included in different studies. The Hakka is a Han ethnic group with a unique genetic background formed by the Hakka ancestors from the Han nationality in central China, who migrated southward for many times and fused with the ancient Yue residents in Guangdong, Fujian, and Jiangxi provinces [25]. To date, there have been no reports on the relationship between *APOE* polymorphisms and the risk of PCAD patients in this population. The purpose of this study was to investigate the relationship between *APOE* rs429358 and rs7412 polymorphisms and PCAD risk.

## Materials and methods

### Study participants and data collection

This study retrospectively analyzed 301 PCAD patients admitted to Meizhou People’s Hospital from November 2019 to August 2023, and 402 individuals without CAD as controls. The diagnostic criteria for CAD: Coronary angiography (CAG) showed that at least one of the main epicardial vessels (including left main branch, anterior descending branch, circumflex branch, and right coronary artery) had a diameter stenosis > 50%, and clinically diagnosed myocardial infarction [26, 27]. Basic information was collected from our hospital’s medical record system, including age, gender, body mass index (BMI), history of smoking, and history of drinking. BMI was divided into three subgroups based on the Chinese criteria [28, 29]: <18.5 kg/m^2^, 18.5–23.9 kg/m^2^, and ≥ 24.0 kg/m^2^. This study was approved by the Human Ethics Committees of Meizhou People’s Hospital, and performed in accordance with the Declaration of Helsinki.

### Determination of serum lipids and *APOE* genotyping

Fasting blood was collected and serum was isolated. Total cholesterol (TC), triglyceride (TG), high-density lipoprotein cholesterol (HDL-C), and low-density lipoprotein cholesterol (LDL-C), Apolipoprotein A1 (Apo-A1), and Apolipoprotein B (ApoB) levels in serum samples were assessed using automatic biochemical analysis system (Olympus AU5400 system, Tokyo, Japan) and corresponding kits.

Genomic DNA was extracted from venous blood collected from EDTA anticoagulant collection vessels using a blood DNA isolation kit (Qiagen GmbH, Germany). The quality and concentration of the DNA were assessed using a Nano-Drop 2000™ spectrophotometer (ThermoFisher Scientific, USA). *APOE* genotype was amplified by PCR - microarray method (Sinochips Bioscience Co., Ltd., China).

### Statistical analysis

All statistical analysis were performed using SPSS statistical software version 26.0 (IBM Inc., USA). Continuous variables were expressed as means ± standard deviations and were compared using either Student’s t-test or the Mann-Whitney U test. Genotype composition ratios and allele frequencies between groups were analyzed with the χ^2^ test. Hardy-Weinberg equilibrium in the patients and controls was evaluated by χ^2^ test.

Individuals with the ɛ2/ɛ4 genotype (*n* = 9; 4 PCAD patients and 5 controls) were excluded from the analysis of the relationship between *APOE* alleles and clinical characteristics of patients because of the opposite effects of the ε2 and ε4 alleles in lipid metabolism [30, 31]. Univariate analysis and multivariate regression logistic analysis were applied to examine the relationship between *APOE* gene polymorphisms and PCAD. *p* < 0.05 was considered to represent statistical significance.

## Results

### Characteristics of subjects

In this study, there were 355(50.5%) males and 348(49.5%) females, and the difference in sex distribution between PCAD patients and controls was not statistically significant (*p* = 0.819). There were 62 (15.4%) cases with BMI < 18.5 kg/m^2^ and 117 (29.1%) cases with BMI ≥ 24.0 kg/m^2^ in controls, while 14 (4.7%) cases BMI < 18.5 kg/m^2^ and 155 (51.5%) cases with BMI ≥ 24.0 kg/m^2^ in PCAD patients. The difference in BMI distribution among the two groups was statistically significant (*p* < 0.001). The differences of TC (5.07 ± 1.23 vs. 4.46 ± 1.13 mmol/L, *p* < 0.001), TG (2.19 ± 1.88 mmol/L vs. 1.52 ± 1.40 mmol/L, *p* < 0.001), LDL-C (2.72 ± 0.82 mmol/L vs. 2.41 ± 0.75 mmol/L, *p* = 0.023), Apo-A1 (1.15 ± 0.26 g/L vs. 1.09 ± 0.33 g/L, *p* = 0.009) and Apo-B (0.88 ± 0.25 g/L vs. 0.76 ± 0.22 g/L, *p* < 0.001) levels among patients and controls were statistically significant. The proportions of PCAD patients with history of smoking (26.9% vs. 7.2%, *p* < 0.001), and history of alcoholism (8.6% vs. 2.2%, *p* < 0.001) were significantly higher than those of controls (Table 1).

**Table 1:** Clinical characteristics of the subjects of this study

Variables	Total (*n* = 703)	Controls (*n* = 402)	PCAD patients (*n* = 301)	*p* values
**Gender**				
Male, n(%)	355(50.5%)	205(51.0%)	150(49.8%)	0.819
Female, n(%)	348(49.5%)	197(49.0%)	151(50.2%)	
**BMI (kg/m** ^**2**^ **)**				
< 18.5	76(10.8%)	62(15.4%)	14(4.7%)	< 0.001
18.5–23.9	355(50.5%)	223(55.5%)	132(43.9%)	
≥ 24.0	272(38.7%)	117(29.1%)	155(51.5%)	
**History of smoking**				
No	593(84.4%)	373(92.8%)	220(73.1%)	< 0.001
Yes	110(15.6%)	29(7.2%)	81(26.9%)	
**History of alcoholism**				
No	668(95.0%)	393(97.8%)	275(91.4%)	< 0.001
Yes	35(5.0%)	9(2.2%)	26(8.6%)	
**Serum lipid levels**				
TC, mmol/L	4.72 ± 1.21	4.46 ± 1.13	5.07 ± 1.23	< 0.001
TG, mmol/L	1.81 ± 1.65	1.52 ± 1.40	2.19 ± 1.88	< 0.001
HDL-C, mmol/L	1.23 ± 0.41	1.24 ± 0.44	1.21 ± 0.36	0.274
LDL-C, mmol/L	2.55 ± 0.80	2.41 ± 0.75	2.72 ± 0.82	< 0.001
Apo-A1, g/L	1.11 ± 0.31	1.09 ± 0.33	1.15 ± 0.26	0.009
Apo-B, g/L	0.81 ± 0.24	0.76 ± 0.22	0.88 ± 0.25	< 0.001

PCAD, premature coronary artery disease; BMI, body mass index; TC, total cholesterol; TG, triglycerides; HDL-C, high-density lipoprotein-cholesterol; LDL-C, low-density lipoprotein-cholesterol; Apo-A1, apolipoprotein A1; Apo-B, apolipoprotein B

### Distribution of the *APOE* genotypes and alleles between the patients and controls

The results of Hardy-Weinberg equilibrium test showed that the *APOE* genotypes in the PCAD patients (*χ*^2^ = 0.801, *p* = 0.938), and controls (*χ*^2^ = 6.333, *p* = 0.176) confirmed to the Hardy-Weinberg equilibrium, respectively. The frequency of the *APOE* ɛ3/ɛ3 genotype was lower (68.1% vs. 75.4%, *p* = 0.034), while the frequency of the *APOE* ɛ3/ɛ4 genotype was higher in the PCAD patients (18.9% vs. 10.2%, *p* = 0.001) than that in controls. The frequency of the ε4 allele was higher (11.1% vs. 7.0%, *p* = 0.007) in the PCAD patients than that in controls (Table 2).

**Table 2:** Distribution frequencies of *APOE* genotypes and alleles in patients and controls

Variable	Genotype/allele	Total (*n* = 703)	Controls (*n* = 402)	PCAD patients (*n* = 301)	χ^2^	*p* values
*APOE* genotype						
	ɛ2/ɛ2	4(0.6%)	2(0.5%)	2(0.7%)	0.085	1.000
	ɛ2/ɛ3	76(10.8%)	46(11.4%)	30(10.0%)	0.389	0.543
	ɛ2/ɛ4	9(1.3%)	5(1.2%)	4(1.3%)	0.010	1.000
	ɛ3/ɛ3	508(72.3%)	303(75.4%)	205(68.1%)	4.535	0.034
	ɛ3/ɛ4	98(13.9%)	41(10.2%)	57(18.9%)	10.954	0.001
	ɛ4/ɛ4	8(1.1%)	5(1.2%)	3(1.0%)	0.093	1.000
*APOE* allele						
	ε2	93(6.6%)	55(6.8%)	38(6.3%)	0.156	0.745
	ε3	1190(84.6%)	693(86.2%)	497(82.6%)	3.500	0.062
	ε4	123(8.7%)	56(7.0%)	67(11.1%)	7.478	0.007
	HWE (χ^2^, *P*)	χ^2^ = 2.135, *p* = 0.711	χ^2^ = 6.333, *p* = 0.176	χ^2^ = 0.801, *p* = 0.938		

HWE, Hardy Weinberg Equilibrium

### Comparison of clinical features and lipid levels among PCAD patients and controls with different *APOE* ε2, ε3, ε4 alleles

Clinical characteristics and serum lipid-lipoprotein levels were compared among PCAD patients and controls carried different *APOE* alleles, respectively. The PCAD patients with ɛ4 allele had higher level in ApoB (0.90 ± 0.20 g/L vs. 0.77 ± 0.31 g/L) than those with ɛ2 allele; PCAD patients with ɛ2 allele had higher TG level (2.94 ± 3.14 mmol/L vs. 2.04 ± 1.69 mmol/L), while had lower LDL-C level (2.46 ± 0.90 mmol/L vs. 2.77 ± 0.83 mmol/L) and Apo-B level (0.77 ± 0.31 g/L vs. 0.90 ± 0.24 g/L) than those with ɛ3 allele (all *p* < 0.05). There were no statistically significant differences in the distributions of gender, BMI, history of alcoholism, and the level of TC and LDL-C among PCAD patients carried *APOE* ɛ2, ɛ3 and ɛ4 alleles, respectively. The controls carried ɛ4 allele had higher LDL-C (2.57 ± 0.77 mmol/L vs. 2.03 ± 0.58 mmol/L) and Apo-B (0.82 ± 0.22 g/L vs. 0.63 ± 0.17 g/L) level than those with ɛ2 allele; controls carried ɛ2 allele had higher HDL-C level (1.42 ± 0.46 mmol/L vs. 1.22 ± 0.44 mmol/L) and lower LDL-C level (2.03 ± 0.58 mmol/L vs. 2.46 ± 0.75 mmol/L) than those carried ɛ3 allele (all *p* < 0.05) (Table 3).

**Table 3:** Clinical characteristics and serum lipid levels of PCAD patients and controls, stratified by *APOE* ɛ2, ɛ3, ɛ4 alleles

Variables	PCAD patients				Controls			
	ε2 (ɛ2/ɛ2 + ɛ2/ɛ3) (*n* = 32)	ε3 (ɛ3/ɛ3) (*n* = 205)	ε4 (ɛ3/ɛ4 + ɛ4/ɛ4) (*n* = 60)	*p* values	ε2 (ɛ2/ɛ2 + ɛ2/ɛ3) (*n* = 48)	ε3 (ɛ3/ɛ3) (*n* = 303)	ε4 (ɛ3/ɛ4 + ɛ4/ɛ4) (*n* = 46)	*p* values
**Gender**								
Male, n(%)	14(43.8%)	108(52.7%)	26(43.3%)	0.371 (χ^2^=2.154)	22(45.8%)	147(48.5%)	32(69.6%)	0.021 (χ^2^=7.582)
Female, n(%)	18(56.3%)	97(47.3%)	34(56.7%)		26(54.2%)	156(51.5%)	14(30.4%)	
**BMI (kg/m** ^**2**^ **)**								
< 18.5	2(6.3%)	10(4.9%)	2(3.3%)	0.327 (χ^2^=4.346)	13(27.1%)	40(13.2%)	9(19.6%)	0.123 (χ^2^=7.555)
18.5–23.9	15(46.9%)	95(46.3%)	20(33.3%)		23(47.9%)	171(56.4%)	27(58.7%)	
≥ 24.0	15(46.9%)	100(48.8%)	38(63.3%)		12(25.0%)	92(30.4%)	10(21.7%)	
**History of smoking, n(%)**								
No	25(78.1%)	141(68.8%)	51(85.0%)	0.036 (χ^2^=6.671)	45(93.8%)	280(92.4%)	43(93.5%)	1.000 (χ^2^=0.157)
Yes	7(21.9%)	64(31.2%)	9(15.0%)		3(6.3%)	23(7.6%)	3(6.5%)	
**History of alcoholism, n(%)**								
No	30(93.8%)	185(90.2%)	56(93.3%)	0.762 (χ^2^=0.544)	46(95.8%)	297(98.0%)	45(97.8%)	0.600 (χ^2^=0.896)
Yes	2(6.3%)	20(9.8%)	4(6.7%)		2(4.2%)	6(2.0%)	1(2.2%)	
**Serum lipid levels**								
TC, mmol/L	5.14 ± 1.76	5.06 ± 1.18	5.08 ± 1.09	0.944	4.17 ± 0.97	4.49 ± 1.18	4.62 ± 0.91	0.115
TG, mmol/L	2.94 ± 3.14*	2.04 ± 1.69^#^	2.26 ± 1.51	0.037	1.26 ± 0.73	1.52 ± 1.50	1.69 ± 1.19	0.323
HDL-C, mmol/L	1.25 ± 0.44	1.21 ± 0.36	1.19 ± 0.30	0.726	1.42 ± 0.46*^,$^	1.22 ± 0.44^#^	1.24 ± 0.42^#^	0.011
LDL-C, mmol/L	2.46 ± 0.90*	2.77 ± 0.83^#^	2.74 ± 0.71	0.138	2.03 ± 0.58*^,$^	2.46 ± 0.75^#^	2.57 ± 0.77^#^	< 0.001
Apo-A1, g/L	1.18 ± 0.26	1.14 ± 0.28	1.15 ± 0.21	0.794	1.22 ± 0.35*	1.07 ± 0.33^#^	1.10 ± 0.32	0.013
Apo-B, g/L	0.77 ± 0.31*^,$^	0.90 ± 0.24^#^	0.90 ± 0.20^#^	0.016	0.63 ± 0.17*^,$^	0.78 ± 0.22^#^	0.82 ± 0.22^#^	< 0.001

CAD, coronary artery disease; BMI, body mass index; TC, total cholesterol; TG, triglycerides; HDL-C, high-density lipoprotein-cholesterol; LDL-C, low-density lipoprotein-cholesterol; Apo-A1, apolipoprotein A1; Apo-B, apolipoprotein B

^#^compared with ɛ2, *p* < 0.05

*compared with ɛ3, *p* < 0.05

^$^compared with ɛ4, *p* < 0.05

### Association of *APOE* gene polymorphisms with PCAD patients

The results of univariate analysis showed that BMI ≥ 24.0 kg/m^2^ (BMI ≥ 24.0 kg/m^2^ vs. BMI 18.5–23.9 kg/m^2^, odds ratio (OR): 2.238, 95% confidence interval (CI): 1.621–3.090, *p* < 0.001), history of smoking (Yes vs. No, OR: 4.736, 95% CI: 3.002–7.469, *p* < 0.001), history of alcoholism (Yes vs. No, OR: 4.128, 95% CI: 1.905–8.948, *p* < 0.001), ɛ3/ɛ4 genotype (ɛ3/ɛ4 vs. ɛ3/ɛ3, OR: 2.055, 95% CI: 1.325–3.187, *p* = 0.001), ε4 allele (ε4 vs. ε3, OR: 1.928, 95% CI: 1.263–2.943, *p* = 0.002), TC level (OR: 1.562, 95% CI: 1.362–1.791, *p* < 0.001), TG level (OR: 1.418, 95% CI: 1.236–1.628, *p* < 0.001), and LDL-C level (OR: 1.648, 95% CI: 1.352–2.008, *p* < 0.001) were significantly associated with PCAD. Multivariate regression logistic analysis showed that BMI ≥ 24.0 kg/m^2^ (BMI ≥ 24.0 kg/m^2^ vs. BMI 18.5–23.9 kg/m^2^, OR: 1.763, 95% CI: 1.235–2.516, *p* = 0.002), history of smoking (Yes vs. No, OR: 5.098, 95% CI: 2.910–8.930, *p* < 0.001), ɛ3/ɛ4 genotype (ɛ3/ɛ4 vs. ɛ3/ɛ3, OR: 2.203, 95% CI: 1.363–3.559, *p* = 0.001), ε4 allele (ε4 vs. ε3, OR: 2.125, 95% CI: 1.333–3.389, *p* = 0.002), and TC level (OR: 1.397, 95% CI: 1.023–1.910, *p* = 0.036) were independent risk factors for PCAD (Table 4).

**Table 4:** Logistic regression analysis of risk factors for PCAD

Variables	Univariate OR (95% CI)	*p* values	Multivariate OR (95% CI)	*p* values
**BMI (kg/m** ^**2**^ **)**				
18.5–23.9	1.000 (reference)	–	1.000 (reference)	–
< 18.5	0.381 (0.205–0.708)	0.002	0.324 (0.163–0.645)	0.001
≥ 24.0	2.238 (1.621–3.090)	< 0.001	1.763 (1.235–2.516)	0.002
History of smoking (Yes/No)	4.736 (3.002–7.469)	< 0.001	5.098 (2.910–8.930)	< 0.001
History of alcoholism (Yes/No)	4.128 (1.905–8.948)	< 0.001	1.685 (0.647–4.389)	0.285
***APOE *** **genotypes**				
ɛ3/ɛ3	1.000 (reference)	–	1.000 (reference)	–
ɛ2/ɛ2	1.478 (0.207–10.577)	0.697	4.406 (0.344–56.436)	0.254
ɛ2/ɛ3	0.964 (0.589–1.578)	0.884	1.273 (0.718–2.256)	0.409
ɛ2/ɛ4	1.182 (0.314–4.456)	0.804	1.289 (0.303–5.489)	0.731
ɛ3/ɛ4	2.055 (1.325–3.187)	0.001	2.203 (1.363–3.559)	0.001
ɛ4/ɛ4	0.887 (0.210–3.752)	0.870	1.294 (0.229–7.321)	0.771
***APOE *** **alleles**				
ε3	1.000 (reference)	–	1.000 (reference)	–
ε2	0.985 (0.609–1.594)	0.952	1.330 (0.758–2.334)	0.320
ε4	1.928 (1.263–2.943)	0.002	2.125 (1.333–3.389)	0.002
**Serum lipid levels**				
TC	1.562 (1.362–1.791)	< 0.001	1.397 (1.023–1.910)	0.036
TG	1.418 (1.236–1.628)	< 0.001	1.152 (0.990–1.341)	0.068
LDL-C	1.648 (1.352–2.008)	< 0.001	1.102 (0.706–1.721)	0.669

BMI, body mass index

## Discussion

The population covered by the onset age of PCAD is the main group of society, which not only has a huge impact on the physical and mental health of patients, but also has a significant impact on the family and social economy of patients. Therefore, we should not only pay attention to the high burden of CVDs to our country, but also pay attention to the loss of production and life ability of young and middle-aged people after CAD, early screening, early identification, early intervention has important significance. In this study, BMI ≥ 24.0 kg/m^2^, history of smoking, *APOE* ɛ3/ɛ4 genotype, and TC level were independent risk factors for PCAD.

ApoE is a major lipid-binding protein that serves as a carrier for chylomicron, HDL-C, LDL-C, and VLDL-C [32]. However, the results on the relationship between *APOE* gene polymorphisms and serum lipid level are not consistent. Rajesh Chaudhary et al. evaluated the effect of ApoE on lipids has shown that carriers of the ε2 allele have lower TC level and higher TG level, while carriers of the ε4 allele have higher TC and LDL levels [33]. Wang et al. reported that ε4 carriers had significantly lower ApoE levels and higher LDL-C, and ApoB levels [34]. *APOE* ε3/ε4 genotype carriers had a significantly higher rate of TC in a Senegalese population [35]. Another study showed that the *APOE* ε4 allele is associated with higher serum lipid levels, whereas the ε2 allele is associated with the lower levels [36]. A Pablos-Méndez et al. reported that the presence of ε2 has been associated with lower LDL-C level but with no influence on the HDL-C level [37]. In this study, the PCAD patients with ɛ4 allele had higher level in ApoB than those with ɛ2 allele; PCAD patients with ɛ2 allele had higher TG level, and lower LDL-C level and Apo-B level than those with ɛ3 allele. In recent years, scholars at home and abroad have realized the importance of PCAD and analyzed the pathogenic factors of PCAD successively, such as *APOE* polymorphisms. Abd El-Aziz TA et al. found that *APOE* ε4 allele may be associated with an increased risk of PCAD [22]. Balcerzyk et al. found that the synergistic effect of the ɛ4 allele with some traditional risk factors (such as smoking, high cholesterol levels) is associated with an increased risk of PCAD [38]. However, in a Slovenian population, *APOE* polymorphisms has not been shown to be an independent risk factor for PCAD [24]. These inconsistent results may be due to differences in the study population and sample size included in different studies. In addition, *APOE* ɛ4/ɛ4 genotype was not associated with the risk of PCAD in this study. It may be related to the low number of individuals carried *APOE* ε4/ε4 genotype.

Moreover, traditional cardiovascular risk factors like smoking, diabetes mellitus, and hypertension are associated with premature atherosclerotic artery disease [39, 40]. Fallahzadeh A et al. considered that smoking is more prevalent in PCAD patients [41]. Wei et al. investigated that smoking showed a positive association with multivessel disease PCAD [42]. Smoking was an independent risk factor for early onset of multiple coronary heart disease in a Chinese population [43]. Smoking was significantly positively correlated with PCAD in an Iran population [44]. Indian researchers have found that smoking, low HDL-C level, and obesity are major risk factors for CAD in young rural Indians [45]. The risk of cardiovascular disease from smoking is largely undisputed.

In addition, Wei et al. investigated that hypertension, diabetes mellitus, and obesity showed a positive association with multivessel disease PCAD [42]. Type 2 diabetes mellitus was an independent risk factor for early onset of multiple CAD in a Chinese population [43]. Through systematic review and meta-analysis, Iranian scholars found that diabetes mellitus, family history of CAD, dyslipidemia, smoking, and hypertension were significantly positively correlated with PCAD in Iranian youth [44]. TIAN R et al. showed that the level of oxidative stress in PCAD patients was significantly higher than that of healthy adults, and their BMI, serum TC, TG, and hypersensitive C-reactive protein (CRP) also showed high levels [46]. Compared with MCAD patients, cardiovascular related risk factors (smoking, type 2 furfururia, abnormal lipid metabolism, hypertension, family history of CAD) were more common in PCAD patients, whereas cardiovascular protective factors (HDL-C levels) were significantly lower [46]. Reynolds HR et al. found complex sex differences between angina pectoris, atherosclerosis, and ischemia [47]. Sex differences in PCAD risk may be related to sex differences in coagulation function, inflammation, and the renin-angiotensin system [48]. In addition, PACD is heterogeneous among different populations, which may be related to their different traditional lifestyles [49]. It can be seen that CAD is influenced by both environmental and genetic factors.

Several studies have also been reported on the relationship between lipid levels and PCAD risk. Sanjeev K Sharma et al. found that high cholesterol was associated with premature CAD [50]. Adeel Khoja et al. suggested that dyslipidaemia was a risk factor for PCAD [51]. Familial hypercholesterolaemia was an independent predictor of PCAD [52–54]. T A Abdel-Aziz et al. found that TC, TG, LDL-C, and HDL-C were independent risk factors for the development of PCAD [55]. And elevated plasma oxidized-low-density lipoprotein (ox-LDL) level was independently associated with the very-early CAD (VECAD) [56]. In this study, TC level was an independent risk factor for PCAD.

The risk factors for PCAD are complex and intertwined with each other. At present, traditional risk factors are mainly intervened in clinical practice, and some emerging risk factors are not included in early prevention. It is believed that with further research on the risk factors of PCAD, early warning of risk factors and early intervention can be carried out to reduce the occurrence of adverse cardiovascular events and reduce the burden on families and society. In this study, BMI ≥ 24.0 kg/m^2^, history of smoking, and *APOE* ɛ3/ɛ4 genotype, and TC level were independent risk factors for PCAD. It suggests that overweight and smoking individuals with *APOE* ɛ3/ɛ4 genotype need to be monitored for PCAD risk. There are some shortcomings in this study. First, this study is a retrospective study, with the possibility of information bias and selection bias, and limited sample size and types of collection indicators, which may reduce the reproducibility of results. Second, this study is a single-center study, and although the data analysis shows that the model has good robustness, it still needs to be verified with external data. Third, this study did not analyze the relationship between *APOE* gene polymorphism and clinical treatment effect of PCAD.

## Conclusion

In summary, *APOE* rs429358 and rs7412 polymorphisms were associated with PCAD susceptibility. Genetic factors and traditional risk factors play a role in the development of PCAD. Generally, BMI ≥ 24.0 kg/m^2^, history of smoking, *APOE* ɛ3/ɛ4 genotype, and TC level were independent risk factors for PCAD. It means that young individuals who are overweight, have a history of smoking, and carried *APOE* ɛ3/ɛ4 genotype need to be aware of the risk of developing PCAD.

## Data Availability

The datasets used and analyzed during the current study available from the corresponding author on request.
